# Effects of a native parasitic plant on an exotic invader decrease with increasing host age

**DOI:** 10.1093/aobpla/plv031

**Published:** 2015-04-02

**Authors:** Junmin Li, Beifen Yang, Qiaodi Yan, Jing Zhang, Min Yan, Maihe Li

**Affiliations:** 1 Zhejiang Provincial Key Laboratory of Plant Evolutionary Ecology and Conservation, Taizhou 318000, China; 2 Institute of Ecology, Taizhou University, Taizhou 318000, China; 3 School of Life Science, Shanxi Normal University, Linfen 041004, China; 4 Ecophysiology Group, Forest Dynamics, Swiss Federal Research Institute WSL, 8903 Birmensdorf, Switzerland

**Keywords:** Defence, deleterious effect, growth, invasive plant, parasitic plant

## Abstract

Understanding changes in the interactions between parasitic plants and their hosts in relation to ontogenetic changes in the hosts is crucial for successful use of parasitic plants as biological controls. We investigated growth, photosynthesis and chemical defences in different-aged *Bidens pilosa* plants in response to infection by *Cuscuta australis*. We were particularly interested in whether plant responses to parasite infection change with changes in the host plant age. Compared with the non-infected *B. pilosa*, parasite infection reduced total host biomass and net photosynthetic rates, but these deleterious effects decreased with increasing host age. Parasite infection reduced the concentrations of total phenolics, total flavonoids and saponins in the younger *B. pilosa* but not in the older *B. pilosa*. Compared with the relatively older and larger plants, younger and smaller plants suffered from more severe damage and are likely less to recover from the infection, suggesting that *C. australis* is only a viable biocontrol agent for younger *B. pilosa* plants.

## Abstract (precis)

Infection by parasitic plants has been considered as an effective method for controlling invasive plants because the parasites partially or completely absorb water, nutrients, and carbohydrates from their host plants, suppressing the vitality of the host. Our study verified that younger and smaller *Bidens pilosa* plants suffer from higher levels of damage and are less likely to recover from infection by the parasitic plant *Cuscuta australis* than relatively older and larger plants, suggesting that *Cuscuta australis* is only a viable biocontrol agent for younger *Bidens pilosa* plants.

## Introduction

A parasitic plant is a type of angiosperm (flowering plant) that directly attaches to another plant via a haustorium (Press 1998). Over 4500 known plant species are parasitic to some extent and acquire some or all of their water, carbon and nutrients from a host (Press 1998; Li *et al*. 2014). Parasitic plants are classified as stem or root parasites including facultative, hemiparasitic and holoparasitic forms (Yoder and Scholes 2010).

Infection by parasitic plants has been considered as an effective method for controlling invasive plants because the parasites partially (hemiparasites) or completely (holoparasites) absorb water, nutrients and carbohydrates from their host plants, suppressing the vitality of the host (Parker *et al.* 2006; Yu *et al.* 2008, 2009; Li *et al.* 2012). For example, the holoparasite *Cuscuta australis*, native to China, can inhibit the growth of *Bidens pilosa*, an invasive plant in China, and thus serve as an effective biological control agent for controlling the invasive *B. pilosa* (Zhang *et al.* 2012, 2013). Compared with the effects of feeding by herbivores, the defence responses of plants infected by parasitic plants have rarely been studied (Runyon *et al.* 2006; Ranjan *et al*. 2014), even though such knowledge is important for the successful use of parasitic plants as enemies against invasive plants.

It has been documented that plant defences to herbivore or pathogen damage vary with a plant's ontogenetic stages (Boege and Marquis 2006; Barton and Koricheva 2010; Tucker and Avila-Sakar 2010; Barton 2013). The ontogenetic patterns of plant defences were found to differ with plant life form (woody, herbaceous and grass, Barton and Koricheva 2010; Massad 2013), growth stage (seedlings, juveniles, mature plants, Barton and Koricheva 2010; Houter and Pons 2012; Barton 2013), development stage (flowering stage, fruiting stage, Tucker and Avila-Sakar 2010) and growth rate (slow-growing plant and fast-growing plant, Massad 2013). For herbaceous plants, for example, young plants are normally more heavily chemically defended than older ones (Cipollini and Redman 1999; Barton and Koricheva 2010; Massad 2013). However, as expected from the resource limitation hypothesis, the smaller reserves of resources stored in younger plants may negatively influence secondary metabolites in comparison with the larger resource reserves stored in mature plants, as expected from the resource limitation hypothesis (Bryant *et al*. 1991). Thus, younger plants may be less defended and less able to recover after herbivory or parasitic infestation (Hódar *et al.* 2008).

Similar to those defence reaction induced by herbivores and pathogens infection, plants may increase their chemical complexes to defend against parasitic infection through plant hormones, salicylic acid and jasmonic acid pathway (Runyon *et al*. 2010). Little attention has been paid to the ontogenetic changes of invasive plants inresponse to holoparasites. Wu *et al.* (2013) found that *Cuscuta campestris* seedlings cannot parasitize the invasive *Mikania micrantha* if the stem diameter of the host is ≥0.3 cm. Our field investigation found that the infestation rate of plants, such as *B. pilosa*, *Solidago canadensis* and *Phytolacca americana*, by *C. australis* decreased with increasing host age (data not shown). Accordingly, we conducted an experiment to understand the host defences in relation to host age in a holoparasite–host system. The growth characteristics and the concentrations of the main chemical defences were determined in different-aged invasive *B. pilosa* plants infected by *C. australis* to test the hypothesis that younger hosts are more easily damaged and less able to recover than the older ones because the younger plants with limited resource reserves have less capacity to produce chemical defences and to promote compensatory growth. We aimed to answer the following questions: (i) Do younger and older *B. pilosa* plants differ in their responses to infection by *C. australis*? (ii) Are these differences in responses are correlated with the growth of different-aged invasive host plants? The answers to these questions could provide basic scientific knowledge for using *C. australis* to manage the invasive plant *B. pilosa*.

## Methods

### Plant species

*Bidens pilosa* is native to the tropical America and has widely spread throughout China. It is an annual forb and can grow up to 1 m in height and produces numerous seeds every year, and it grows both in nutrient-rich and -poor soils. In November, 2009, seeds of *B. pilosa* were collected near Sanfeng temple (121°16′E, 28°88′N) in Linhai City, Zhejiang Province, China, and stored in a low-humidity storage cabinet (HZM-600, Beijing Biofuture Institute of Bioscience and Biotechnology Development) until use.

*Cuscuta australis*, a native annual holoparasitic plant species to South China, and is considered a noxious weed of agriculture (Yu *et al.* 2011). It can infect a wide range of herbs and shrubs (e.g. plants in the families of Fabaceae and Asteraceae), including the invasive plants *M. micrantha*, *Ipomoea cairica*, *Wedelia trilobata*, *Alternanthera philoxeroides* and *Bidens* (Yu *et al.* 2011; Wang *et al.* 2012; Zhang *et al.* 2012).

### Experimental design

We conducted a greenhouse experiment at Taizhou University (121°17′E, 28°87′N) in Linhai City, Zhejiang Province, China. We sowed *B. pilosa* seeds in trays with sand to germinate in a greenhouse on 13 March, 22 March and 6 April 2011, to create three different-aged *B. pilosa* seedlings of three different ages. Approximately 20 days after sowing, *B. pilosa* seedlings (∼10 cm in height) were transplanted into pots (28 cm in inner diameter and 38 cm deep; 1 seedling per pot) filled with 2.5 kg yellow clay soil mixed with sand in a 2 : 1 ratio (v : v). Plant materials and stones were removed from the yellow clay soil collected from fields in Linhai. The soil mixture had a pH of 6.64 ± 0.01, with an organic matter content of 15.74 ± 2.65 g kg^−1^, available nitrogen of 0.27 ± 0.10 g kg^−1^, available phosphorus of 0.026 ± 0.004 g kg^−1^ and available potassium of 0.049 ± 0.003 g kg^−1^.

The pots were randomly placed in a greenhouse and irrigated with tap water twice daily. One week after transplantation, 2 g slow release fertilizer (Scotts Osmocote, N : P : K = 20 : 20 : 20, The Scotts Miracle-Gro Company, Marysville, OH, USA) was added to each pot.

On 5 June, when *B. pilosa* plants were of ages 59 days (mean height 32.0 cm and mean diameter 2.7 mm), 74 days (mean height 62.6 cm and mean diameter 5.1 mm) and 83 days old (mean height 93.3 cm and mean diameter 5.7 mm), plants were infected by *C. australis* manually. Three 15-cm long segments of parasitic *C. australis* stems collected from fields in Linhai were twined onto the stems of a *B. pilosa* plant to induce infection. After 24 h, most of *C. australis* successfully parasitized the host and died segments were substituted by new ones. For each age class, six individuals were infected and six plants were left intact as controls (*n* = 6). Six individuals were harvested, separated into shoots and roots, and then dried at 70 °C for 72 h, to determine the initial plant biomass (*W*_1_) at the beginning of infection (*t*_1_, i.e. 5 June).

### Measurements

On 30 June 2011, i.e. after 26 days of infection, the net photosynthetic rate (*P*_n_) of *B. pilosa* plants was determined on fully expanded, mature sun leaves in the upper canopy between 10:00 and 11:30 am, using a portable photosynthesis system (LI-6400/XT, LI-COR Biosciences, Lincoln, NE, USA). For each measurement, three leaves per plant were chosen, and six consecutive measurements were performed.

On 9 July 2011 (*t*_2_), i.e. 35 days after infection, when *C. australis* was flowering and the host plants were 94, 109 and 118 days old, respectively, all plants were harvested. *Cuscuta australis* plants were separated from their hosts and dried at 70 °C for 72 h to determine the *C. australis*' biomass (*B*_c_). The host plants were separated into leaves, stems and roots. Leaves, stems and roots of the host plants were dried at 70 °C for 72 h to determine their biomass (*W*_2_). The relative growth rate (RGR) of biomass was calculated with the equation RGR = (ln *W*_2_ − ln *W*_1_)/(*t*_2_ − *t*_1_) (González-Santana *et al*. 2012; Li *et al.* 2012).

The dried stems of the host plants were ground using a universal high-speed grinder (F80, Xinkang Medical Instrument Co. Ltd, Jiangyan, Jiangsu). The powder was filtered through a 20-mesh sieve and stored in a drier until chemical analysis.

Approximately 0.1 g of powder was extracted three times with 70 % ethanol (v/v) under reflux at 90 °C and the aqueous extract was used to measure the concentration of total phenolics and total flavonoids. The concentration of total phenolics and total flavonoids was determined using the Folin–Denis method and AlCl_3_ reaction method according to Cortés-Rojas *et al*. (2013) and Jin *et al.* (2007). Absorbance at 750 nm for total phenolics and 420 nm for total flavonoids was determined with a T6 UV–VIS spectrophotometer (Beijing Purkinje General Instrument Co. Ltd, Beijing, China). Gallic acid and rutin (purchased from National Institutes for Food and Drug Control, Beijing, China) were used as the standard for total phenolics and total flavonoids, respectively.

Approximately 0.1 g of powder was extracted three times with 70 % methanol under reflux at 70 °C, and the aqueous extract was used to measure the concentration of saponins and alkaloids. The concentration of total saponins and alkaloids was determined by a colourimetric method and bromocresol green reaction method, respectively, according to Li *et al*. (2006) and Jin *et al*. (2006). Absorbance at 560 nm (saponins) and 470 nm (alkaloids) was determined with a T6 UV–VIS spectrophotometer (Beijing Purkinje General Instrument Co. Ltd, Beijing, China). Ginsenosides-Re and berberin HCl (purchased from National Institutes for Food and Drug Control, Beijing, China) were used as the standard for saponins and alkaloids, respectively.

Approximately 0.1 g of powder was extracted three times with boiled water and the aqueous extract was used to measure the concentration of soluble tannin via the potassium permanganate redox titrations method according to Li *et al*. (2007). Gallic acid (purchased from National Institutes for Food and Drug Control, Beijing, China) was used as the standard.

### Data analysis

The plastic responses (PR) of the host plants to parasite infection were calculated for all plant traits studied, using the equation PR = (VPP − MVC)/MVC (Barton 2008), where VPP is the value of a trait in a parasite-infected plant and MVC is the mean value of that trait in the same-aged controls. For example, if the mean biomass of the non-parasitized *B. pilosa* is *A* and the biomass of the parasitized *B. pilosa* is *B*, then PR = (*B* − *A*)/*A*. Such PR values reflect the relative changes in the host traits caused by parasites. A value of PR = 0 indicates no response. A value of PR < 0 indicates a negative response, whereas a value of PR > 0 indicates a positive response of the host to parasite infection.

The normality of the distribution and the homogeneity of the data were checked (Kolmogorov–Smirnov test) before any further statistical analysis. A two-way ANOVA was used to analyse the effects of parasitism and host age on the host traits studied, followed by a one-way ANOVA and Tukey's HSD analysis to test the difference in means within a parasite treatment and between the infected and non-infected plants. All tests were conducted at a significance level of *P* < 0.05 using SPSS (version 16.0).

## Results

### Effects of *C. australis* infection on host growth

**Figure 1.**
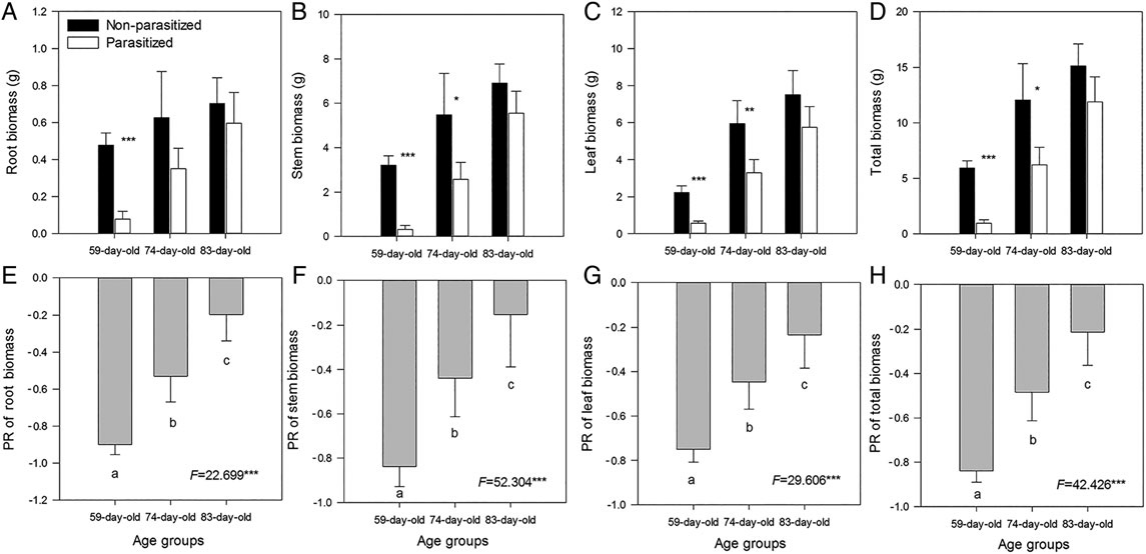
The root (A), stem (B), leaf (C) and total plant biomass (D) of different-aged invasive *B. pilosa* plants infected and not infected by *C. australis*, and the PR of the stem (E), root (F), leaf (G) and total plant biomass (H) of the infected *B. pilosa* plants. Values are given as means +1 SD (*n* = 6). Asterisks in the upper panel indicate significant difference in means between non-infected and infected plants within the same age class at *P < 0.05, **P < 0.01 and ***P < 0.001, respectively. Different letters in the lower panel indicate significant difference between PRs (*P* < 0.05).

*Cuscuta australis* infection decreased the root, stem, leaf and total biomass of the hosts compared with the controls within each age class (Fig. 1, upper panel), and this negative effect on host growth significantly decreased with increasing host age (Fig. 1, lower panel). Compared with the non-infected control hosts, the total plant biomass of the infected hosts decreased 84 % for the 59-day-old plants, 48 % for the 74-day-old plants and 21 % for the 83-day-old plants.

**Figure 2.**
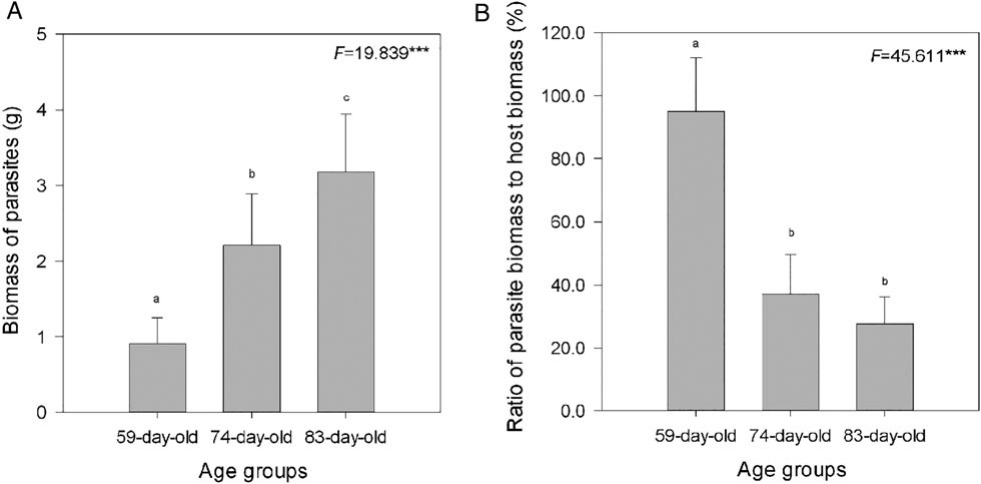
The biomass of parasites (A) of different-aged invasive *B. pilosa* plants, and the ratio of parasite biomass to host biomass (B). Values are given as means +1 SD (*n* = 6). Different letters indicate significant difference between host plants of different ages at *P* < 0.05. *F*-value and significance levels are given. ***Significant difference in means between plants within the same age class at *P* < 0.001.

The growth of the parasites significantly increased with host age (Fig. 2A). However, the biomass ratio of parasite to host significantly decreased with host age (Fig. 2B).

**Figure 3.**
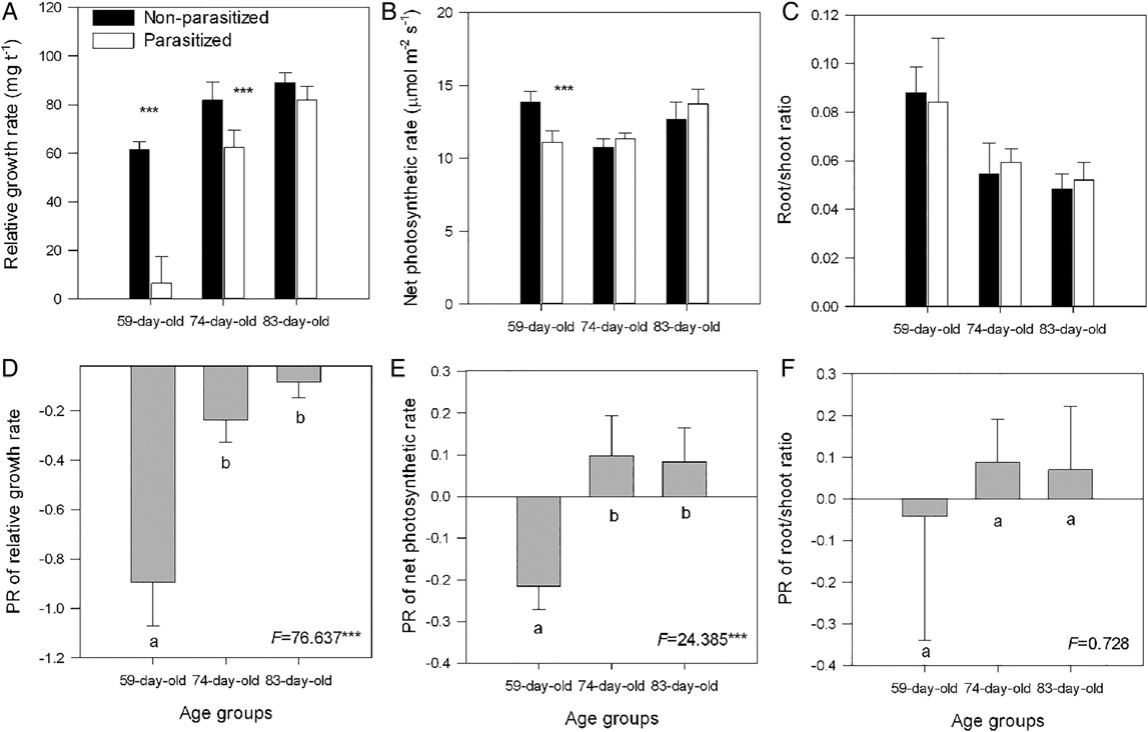
The RGR (A), net photosynthetic rate of leaves (B) and root/shoot ratio (C) of different-aged invasive *B. pilosa* plants infected and not infected by *C. australis*, and the PR of RGR of plant biomass (D), net photosynthetic rate of leaves (E) and ratio of root biomass to shoot biomass (F) of different-aged invasive *B. pilosa* to the parasitic *C. australis*. Values are given as means +1 SD (*n* = 6). Different letters indicate significant difference between host plants of different ages at *P* < 0.05. *F*-value and significance levels are given. Asterisks indicate significant difference in means between plants within the same age class at ***P < 0.001.

The infection of *C. australis* significantly decreased the growth rates in the younger (i.e. the 59- and 74-day-old hosts; both *P* < 0.001) but not in the 83-day-old hosts compared with those in the corresponding controls (Fig. 3A and D). The infection significantly suppressed the net photosynthetic rates only in the younger (59- and 74-day-old) hosts but not in the older hosts (Fig. 3B and E). *Cuscuta australis* infection had no effects on the root/shoot ratio in different-aged hosts (Fig. 3C), and the root/shoot ratio tended to decrease with increasing host age (Fig. 3C). The negative effect of *C. australis* infection on *B. pilosa*'s RGRs (Fig. 3D), net photosynthetic rates (Fig. 3E) and root/shoot ratios (Fig. 3F) decreased with increasing host age (Fig. 3D–F). Host age (*A*) interacted with parasites (*P*) to affect the total biomass (*P* < 0.01 for *A* × *P* interaction), RGRs (*P* < 0.001) and net photosynthetic rates (*P* < 0.001) of the hosts (Table 1).

**Table 1.** Two-way ANOVA analysis of age and parasitism on the growth of invasive *B. pilosa*. Bold values indicate significant effects on the growth of *B. pilosa*.

	df	Leaf biomass		Stem biomass		Root biomass		Total biomass		Root/shoot ratio		Relative growth rate		Net photosynthetic rate	
		*F*	*P*	*F*	*P*	*F*	*P*	*F*	*P*	*F*	*P*	*F*	*P*	*F*	*P*
Age (*A*)	2	97.135	**<0**.**001**	59.992	**<0**.**001**	19.819	**<0**.**001**	121.986	**<0**.**001**	23.926	**<0**.**001**	175.610	**<0**.**001**	29.308	**<0**.**001**
Parasitism (*P*)	1	43.267	**<0**.**001**	51.293	**<0**.**001**	29.009	**<0**.**001**	116.349	**<0**.**001**	0.112	0.740	137.485	**<0**.**001**	131.144	**<0**.**001**
*A* × *P*	2	1.036	0.367	2.368	0.111	3.065	0.061	9.763	**<0**.**01**	0.342	0.713	38.004	**<0**.**001**	50.772	**<0**.**001**

### Effects of infection on host's secondary metabolites

**Figure 4.**
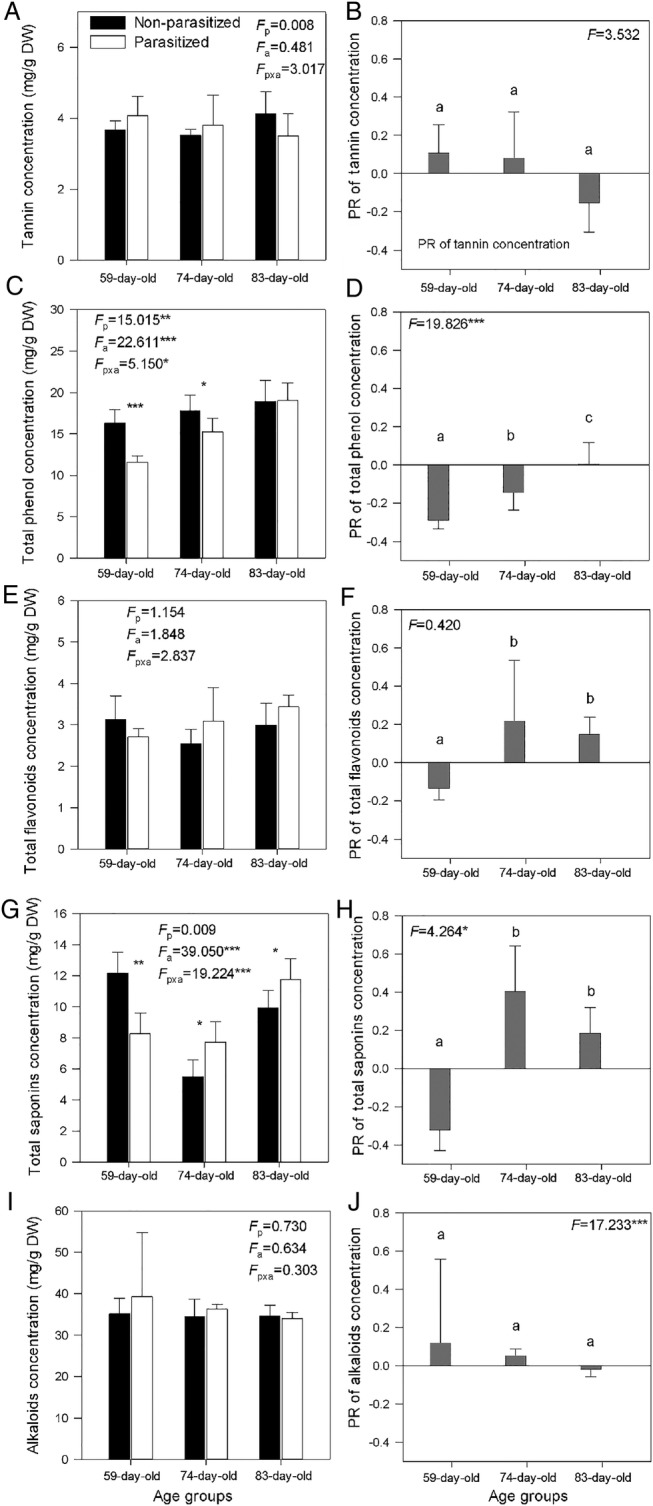
The concentrations (mean values + 1 SD, *n* = 6) of tannin (A), total phenolics (C), total flavonoids (E), saponins (G) and alkaloids (I) in different-aged invasive *B. pilosa* plants infected and not infected by *C. australis*, and the PR of tannin (B), total phenolics (D), total flavonoids (F), saponins (H) and alkaloids (J) in stems of the different-aged *B. pilosa* plants to the parasitic *C. australis*. Different letters indicate significant difference between host plants of different ages at *P* < 0.05. *F*-value and significance levels are given. Asterisks indicate significant difference in means between plants within the same age class at *P <0.05, **P <0.01 and ***P < 0.001, respectively.

The infection significantly decreased the concentrations of the phenolics in the younger plants (59 and 74 days old) but not in the older hosts (Fig. 4C), and the negative effect of *C. australis* decreased with increasing host age (Fig. 4D). The infection significantly decreased the concentrations of the terpenoids in the younger plants (59 days old) but significantly increased those concentrations in the older plants (74 and 83 days old) (Fig. 4G). Host age interacted with parasite infection to influence the levels of total phenols and saponins (Fig. 4C and G). No effects of host age, parasite infection and their interaction on the concentrations of tannin, total flavonoids and alkaloids in the hosts were observed (Fig. 4A, E and I). The effect of infection on the concentration of total flavonoids in the younger plants was negative (59 days old), whereas that in the older plants was positive (74 and 83 days old) (Fig. 4F).

## Discussion

### Recovery ability in relation to the host age

The present study found that the deleterious effects of parasite infection on the older *B. pilosa* were significantly less severe than on the younger plants, indicating that the younger plants were more sensitive to parasite infection than the older ones. These results supported our hypothesis that the damage to younger *B. pilosa* caused by *C. australis* infection is greater than the damage to older *B. pilosa*.

Previous studies have shown that older hosts exhibit a defence mechanism that hampers the development of haustoria and thus mitigates parasite infection (Runyon *et al*. 2006; Meulebrouck *et al.* 2009; Lee and Jernstedt 2013). However, this conclusion was not supported by the present study because the parasite biomass did not decrease but increased with increasing host age in association with host size. Older hosts, with larger size and greater resource storage, could support greater growth of the attached parasites, leading to a mean increase in parasite biomass by 142 % for the 74-day-old hosts and 248 % for the 83-day-old hosts compared with that for the 59-day-old hosts.

Our study found that root, stem, leaf and total biomass, RGR and photosynthetic ability were significantly negatively affected by parasite infection in the younger plants (59 and 74 days old) but not in the older plants (83 days old), indicating that the young plants fail to compensate whereas the older plants do (Tan *et al.* 2004; Zhang *et al*. 2012). Herbivory can induce compensatory growth by stimulating photosynthesis, altering mass allocation and increasing growth rates (Markkola *et al*. 2004; Hódar *et al.* 2008). Therefore, the responses of younger *B. pilosa* to parasite infection is not similar to the responses of plants to damage by herbivores (Barton and Koricheva 2010; Barton 2013). Stout *et al.* (2002) have found that younger rice plants appeared to be less tolerant to herbivory than older rice plants, though Elger *et al.* (2009) have reported a higher sensitivity to herbivore attacks in young seedlings of British grassland species than in older conspecifics.

Parasite infection had no effects on the root/shoot ratio in different-aged hosts, i.e. parasite infection did not alter the mass allocation to roots and shoots. This result may be due primarily to changes in the light conditions caused by the attack behaviour of the parasite. Herbivores destroy parts of plants, resulting in increases in light quality and quantity within a plant and thus leading to increases in photosynthesis and growth rates. In contrast, parasite infection may shade a host and decrease the light intensity within a host plant, especially if the hosts plants are small or young, thus leading to decreases in photosynthesis and growth rates (Rijkers *et al.* 2000).

The decreases in photosynthesis induced by parasitic infection led to a limited availability of resources, which further resulted in lower growth rates in smaller and younger hosts. These results indicated that the resistance of hosts to parasitic infection is negatively correlated with the availability of the resources stored in a host plant (Shen *et al*. 2013). For woody forest plants, negative effects of mistletoe infection on host tree growth and mortality have been consistently and extensively reported (Shaw *et al.* 2008; Logan *et al*. 2013), and those negative growth effects have widely been considered to result from decreased photosynthetic production (Meinzer *et al.* 2004) caused by decreased leaf size and leaf N content (Ehleringer *et al.* 1986; Cechin and Press 1993; Logan *et al.* 1999; Mishra *et al*. 2007).

### Chemical defence relative to host age

Little attention has been paid to changes in chemical defences, such as alkaloids, phenolics, flavonoids, cyanogenic glycosides (Elger *et al*. 2009; Quintero and Bowers 2013), induced by parasitic infection, whereas ontogenetic changes in chemical defences against herbivory are well documented (Van Zandt and Agrawal 2004; Barton 2008). The concentrations of cyanogenic glycoside (Schappert and Shore 2000), nicotine (Ohnmeiss and Baldwin 2000), alkaloids (Ohnmeiss and Baldwin 2000; Elger *et al*. 2009) and phenolics (Donaldson *et al*. 2006; Elger *et al*. 2009) have been found to be higher in older tissues/plants than in younger tissues/plants exposed to herbivory. The present study found that the concentrations of total phenolics, total flavonoids and saponins were significantly negatively affected by *C. australis* infection in younger *B. pilosa* but not in older plants. A positive response of total phenolics was found only in the 83-day-old plants; a positive response of total flavonoids and saponins occurred in both 74- and 83-day-old plants. These results supported our initial hypothesis that the younger plants with limited resource reserves have less leeway to produce chemical defences. Similarly, it has been reported that older plants that had accumulated resources over a long period were better able to maintain anti-herbivore defences than younger plants with limited resources (Boege 2005; Elger *et al*. 2009).

However, other studies have reported opposite or neutral responses of chemical defences to herbivory (Thomson *et al.* 2003; Barton and Koricheva 2010). Goodger *et al.* (2013) demonstrated that the levels of phenolics produced in response to herbivory were highest in seedlings compared with those in juveniles and mature trees. A meta-analysis based on data from 36 published studies also did not find any clear relationships between ontogenetic stage and chemical defences induced by herbivory (Barton and Koricheva 2010). Ontogenetic patterns of plant chemical responses to herbivory or parasite infection might vary with plant species, chemical compounds and disturbance (e.g. parasite or insect infection) (Webber and Woodrow 2009; Rohr *et al*. 2010). Further studies are needed to identify, clarify and quantify the chemical defences in different hosts and different-aged hosts infected by different parasites.

Plant resistance traits, including physical defences traits and chemical secondary compounds, and tolerance traits such as compensatory re-growth are detectable, when plants are damaged by insect, pathogen or parasite infection (Stowe *et al.* 2000; Quintero and Bowers 2013). Previous studies prove that trade-offs between resistance and tolerance traits of plants do occur following damage (Orians *et al.* 2010; Quintero and Bowers 2013). However, several studies reported that the trade-offs between resistance and tolerance traits are not detectable for all development stages (e.g. Quintero and Bowers 2013), for example, during the seedling development stage (Barton 2008). Our results showed that younger hosts had higher concentrations of tannin and alkaloids but lower concentrations of flavonoids, phenolics and terpenoids, as well as a lower re-growth ability; conversely, older hosts had lower concentration of tannin and alkaloids, but higher concentrations of flavonoids, phenolics and terpenoids, as well as a higher capability of compensatory growth. These results provided an indirect evidence for supporting the above-mentioned viewpoints that younger hosts with lower biomass invest fewer resources in growth but more resources in higher concentrations of tannin and alkaloids, whereas the older hosts have a higher biomass and higher concentrations of flavonoids, phenolics and terpenoids, but lower concentrations of tannin and alkaloids. Accordingly, the overall pattern of response might depend on the age or size of the host and even on the diversity of damage.

In conclusion, the intensity of damage caused by the parasite *C. australis* was dependent on the host age or size associated with the host biomass, showing that younger and smaller hosts were more easily damaged and less able to recover. This result supported the resource limitation hypothesis (Bryant *et al*. 1991) because hosts with increasing host age (or size) have more resources available to defend against or limit the establishment of haustoria (Runyon *et al.* 2010). Our field investigation found that *B. pilosa* plants with a height >90 cm were resistant because they were less infected by *C. australis*, whereas *B. pilosa* plants with a height <30 cm were effectively and successfully controlled by *C. australis*. Our results, therefore, indicate that the parasite *C. australis* could be successfully used as a potential biological agent in the control of invasive *B. pilosa* plants only in the early stages of development. However, as Boege (2005) indicated, further studies are needed to better and fully understand the mechanisms underlying the effects of host ontogeny on the responses of the host plants to herbivores or (holo) parasites.

## Sources of Funding

This work was financially supported by the National Natural Science Foundation of China (No. 31270461; No. 30800133) and National Natural Science Foundation of Zhejiang Province (No. Y5110227).

## Contributions by the Authors

J.L. and M.Y. conceived and designed research. B.Y., Q.Y. and J.Z. conducted experiments. J.L. analysed data. J.L. and M.L. wrote the manuscript. All authors read and approved the manuscript.

## Conflict of Interest Statement

None declared.
